# HOXA13, Negatively Regulated by miR-139-5p, Decreases the Sensitivity of Gastric Cancer to 5-Fluorouracil Possibly by Targeting ABCC4

**DOI:** 10.3389/fonc.2021.645979

**Published:** 2021-05-21

**Authors:** Zhengqian Chen, Zhiwei Qin, Lei Li, Qi Wo, Xia Chen

**Affiliations:** ^1^ Department of Breast Surgical Oncology, Fujian Medical University Cancer Hospital & Fujian Cancer Hospital, Fuzhou, China; ^2^ Department of General Surgery, Shanghai General Hospital, Shanghai Jiao Tong University School of Medicine, Shanghai, China

**Keywords:** chemoresistance, gastric cancer, HOXA13, ABCC4, miR-139-5p

## Abstract

**Purpose:**

Chemoresistance remains a major challenge in the therapy of gastric cancer (GC). The homeobox (HOX) gene family has gained attention in carcinogenesis and chemoresistance. Here, this study aimed to explore the mechanism of HOXA13 in GC chemoresistance.

**Methods:**

Quantitative real-time PCR (qRT-PCR) and Western blot were used to evaluate the expression of HOXA13 in GC tissues. The Kaplan–Meier plotter database was mined for prognosis analysis of GC patients with different HOXA13 expression receiving 5-Fluorouracil (5-FU) therapy. The effects of HOXA13 on sensitivity of GC cells to 5-FU were investigated by Cell Counting Kit-8 (CCK-8), 5-Ethynyl-2’-deoxyuridine (EdU) incorporation, flow cytometry and experiment *in vivo*. RNA-Sequencing analysis was performed to explore the underlying mechanism of HOXA13-mediated 5-FU resistance in GC. Chromatin immunoprecipitation (ChIP) and rescue experiments were applied to determine the relationship between HOXA13 and ABCC4. Luciferase reporter assay was performed to assess interaction of miR-139-5p and HOXA13.

**Results:**

HOXA13 was upregulated in GC and its high expression was associated with poor prognosis of GC patients with 5-FU treatment. Overexpression of HOXA13 impaired the inhibitory effects of 5-FU on GC cells proliferation *in vitro* and *vivo*, and knockdown of HOXA13 exacerbated 5-FU-induced GC cells apoptosis. Mechanistically, HOXA13, directly targeted by miR-139-5p in GC, might upregulate ABCC4 expression, thereby accentuating 5-FU resistance of GC cells.

**Conclusion:**

Our study suggests that HOXA13 attenuates 5-FU sensitivity of GC possibly by upregulating ABCC4. Thus, targeting HOXA13 would provide a novel prospective into the potential therapeutic strategy for reversing chemoresistance.

## Introduction

Gastric cancer (GC) is one of the most common malignancies and faces high risk of fatality worldwide, especially in East Asia (1). Chemotherapy has been identified as one of the typical treatments for GC for decades. 5-Fluorouracil (5-FU), which is the most commonly administrated anti-cancer agent in GC, has noteworthily improved survival in patients with advanced GC (2, 3). However, the emergence of drug resistance turns out to be a major challenge to treatment efficacy, particularly in patients with recurrence and metastasis (4). Thus, probing into the underlying mechanisms and potential targets of chemoresistance of GC is crucial and could further facilitate ameliorating the prognosis of GC patients.

Homeobox (HOX) genes constitute a set of transcription factors that are essential for embryonic development and their dysregulation is involved in the tumorigenesis and chemosensitivity of multiple cancers (5–9). Recently, the role of HOXA13, a member of HOX family, in carcinogenesis and chemotherapy resistance has attracted increasing attention. For instance, the high HOXA13 expression in hepatocellular carcinoma (HCC) is associated with patients’ clinical progression and predicts disease outcome (10). Downregulation of HOXA13 inhibits cell proliferation and chemoresistance in small cell lung cancer (11). Upregulation of HOXA13 promotes resistance to gemcitabine of pancreatic ductal adenocarcinoma (PDAC) cells (12). While the significant role HOXA13 plays in various cancers, the specific mechanism of HOXA13 in GC chemoresistance remains to be further explored.

ATP-binding cassette (ABC) transporters, a group of membrane protein complexes, are divided into seven subfamilies, ABCA through ABCG (13). ABCC-subfamily (the multidrug resistance-associated proteins, MRPs), the main branch of ABC transporters, has been proven to actively pump drugs out of tumor cells, thereby avoiding the cytotoxicity of chemotherapeutics (14). Recently, many studies have illustrated the relationship between ATP-binding cassette subfamily C member 4 (ABCC4) and tumor chemoresistance. Gazzaniga et al. demonstrated that ABCC4 enhances resistance to multiple chemotherapeutic drugs in metastatic breast cancer (15). In addition, inhibiting the expression of ABCC4 sensitizes neuroblastoma to irinotecan (16).

Our previous study indicated that HOXA13 was upregulated in GC tissues and promoted proliferation and metastasis in GC cells (17). In this study, we found that high expression of HOXA13 was in association with poorer 5-FU treatment response in GC. It showed that HOXA13 overexpression increased 5-FU resistance in GC cells, while HOXA13 knockdown led to the opposite results. HOXA13 impaired the anti-proliferative effect of 5-FU and suppressed 5-FU-induced apoptosis. Mechanistically, we demonstrated that HOXA13 upregulated ABCC4 expression *via* binding to its promoter region, which was further testified to reverse HOXA13-induced 5-FU resistance in GC cells. Inquiring the probable regulation mechanism of HOXA13, bioinformatics analysis and experimental verification revealed that HOXA13 was directly targeted by miR-139-5p. Together, these results indicated that HOXA13 played an indispensable part in 5-FU chemoresistance in GC, during which process ABC transporters activation, especially ABCC4 upregulation, might serve as one of the essential downstream signal transduction mechanisms.

## Material and Methods

### Patients and Tissue Samples

Forty-two pairs of GC tissues and matched normal tissues were collected from patients undergoing GC resection at Shanghai General Hospital (Shanghai, China). The samples were obtained from the patients with informed consent. The study was approved by the Ethics Committee of Shanghai General Hospital.

### Cell Lines and Cell Culture

The human gastric cancer cell lines (AGS, MKN28, MKN45, SGC7901) and normal human gastric epithelial cells-1 (GES-1) were preserved by the General Surgery Institute, Shanghai General Hospital. Cells were cultured in RPMI-1640 medium containing 10% fetal bovine serum (Gibco, California, USA) and 1% penicillin–streptomycin. Cells were maintained at 37°C in a humidified atmosphere with 5% CO_2._

### Lentiviral Transduction and Transient Transfection

The HOXA13 lentiviral vector and HOXA13 shRNA lentiviral vector were supplied by Genomeditech (Shanghai, China). Lentivirus were transfected into GC cells and then stable transfected cells were selected with puromycin. Also, stable cell lines with luciferase were selected by blasticidin according to manufacturer’s instructions. The cell lines were divided into the following categories: Vector, infected with the lentiviral vector containing the control fragment; HOXA13, infected with the lentiviral vector containing the HOXA13 fragment; shNC, infected with the control shRNA lentivirus; shHOXA13, infected with Lenti-shRNA. In rescue experiments, cells were transiently transfected with siRNA targeting ABCC4 (Genomeditech) or ABCC4-overexpressing plasmid (NovaBio, Shanghai, China) using Lipo3000 (Invitrogen, California, USA).

### Quantitative Real-Time PCR (qRT-PCR)

Total RNA was extracted from tissues and cells using TRIzol (Takara, Shiga, Japan) according to the manufacturer’s instructions. RNA was reverse transcribed to cDNA using the Hifair™ First Strand cDNA Synthesis SuperMix (Yeasen, Shanghai, China). Quantitative real-time PCR was performed using the Hifair™ qPCR SYBR Green Master Mix (Yeasen) in three technical replicates. The expression values of indicated genes were normalized to GAPDH and calculated using the 2^−ΔΔCt^ method. The primers were listed below: GAPDH F: 5’-GGGAAGGTGAAGGTCGGAGT-3’, R: 5’-GGGGTCATTGATGGCAACA-3’; HOXA13 F: 5’-GAACGGCCAAATGTACTGCC-3’, R: 5’-GTATAAGGCACGCGCTTCTTTC-3’; ABCC4 F: 5’-GCAAAATCATCGTGTTTGTGAC-3’, R: 5’-AAAAGGTCTGGATTCTTCGGAT-3’.

### Western Blot Analysis

Total proteins from tissues and cells were extracted using RIPA lysis buffer with 1% protease and phosphatase inhibitor cocktail (NCM, Jiangsu, China). Cell lysates were separated by SDS-PAGE and transferred onto PVDF membranes. After blocking with protein free rapid blocking buffer (EpiZyme, Shanghai, China), the membranes were incubated with primary antibodies at 4°C overnight. The next day, the membranes were washed and incubated with HRP-conjugated goat anti-rabbit or anti-mouse secondary antibody (Cell Signaling Technology, MA, USA). Then protein bands were visualized using ECL chemiluminescent reagent (Millipore, MA, USA). The antibodies used in this study included anti-HOXA13 (1:1,000; Abcam, Cambridge, UK), anti-ABCC4 (1:1,000; Santa Cruz, CA, USA), anti-tubulin (1:1,000; Cell Signaling Technology), anti-cleaved caspase-3 (1:1,000; Cell Signaling Technology), anti-cleaved caspase-9 (1:1,000; Cell Signaling Technology), anti-MDM2 (1:1,000; Cell Signaling Technology), and anti-p53 (1:1,000; Abcam, Cambridge, UK).

### The Kaplan–Meier Plotter

Survival analyses based on HOXA13 and ABCC4 expression level in GC were analyzed from the Kaplan–Meier plotter (http://kmplot.com/analysis/) (18). The GC cases with their acceptance of 5-FU were divided into two cohorts according to the auto select best cutoff. Overall survival (OS) and post progression survival (PPS) of GC patients in different groups were assessed by the Kaplan–Meier plot with hazard ratio (HR) and log-rank P value.

### Drug Sensitivity Assay

To evaluate the toxicity of 5-FU in cells, GC cells were seeded into each well of 96-well plates and cultured at 37°C for 24 h. Cells were treated with graded concentrations of 5-FU for 48 h. Then 10 μl of Cell Counting Kit-8 (CCK-8) solution (Dojindo, Kumamoto, Japan) was added to each well. The absorbance at 450 nm was measured using a Gen5 microplate reader (BioTek, Vermont, USA). The experiment was tested in three technical replicates.

### 5-Ethynyl-2’-Deoxyuridine (EdU) Staining and Colony Formation Assays

The effect of HOXA13 on cell proliferation upon 5-FU treatment was determined by EdU incorporation assay (RiboBio, Guangdong, China). In brief, cells (1 × 10^4^) were seeded into each well of 96-well plates. After 24 h, cells were cultured in medium supplemented with or without 5-FU for 48 h. Then, medium containing EdU was added for 2 h. The cells were fixed with methanol and stained according to manufacturer’s instructions. Cell proliferation was observed using a fluorescence microscope (DMI6000B, Leica, Germany).

For colony formation assay, cells (1 × 10^3^) were plated in each well of 6-well plates and incubated in medium supplemented with or without 5-FU. After two weeks, colonies were fixed with methanol and dyed with 0.1% crystal violet. Then the colonies were counted.

Each experiment was performed in three technical replicates.

### Apoptosis Assay

Cell apoptosis was performed by using the Annexin V-PE/7-AAD apoptosis kit (MultiSciences, Zhejiang, China). After treatment with or without 5-FU for 48 h, cells were harvested in PBS, and then approximately 5 × 10^5^ cells were resuspended in 500 μl 1× binding buffer and mixed with 5 μl Annexin V-PE and 10 μl 7-AAD for 5 min. The stained cells were analyzed by flow cytometry (Accuri C6, BD Biosciences, USA). The experiment was performed in three technical replicates.

### RNA Sequencing Analysis

AGS-HOXA13 and AGS-Vector cells were treated with 5-FU for the indicated time and total RNA was extracted using TRIzol reagent. The integrity of the purified RNA was analyzed by the 2200 Electrophoresis Bioanalyzer System (Agilent, CA, USA). RNA with RIN (RNA integrity number) >6.0 was considered acceptable for cDNA library construction. Genes were considered significantly differentially expressed under the following criteria using DESeq2: Fold change >1.5, P <0.05. The analysis was performed in three biological replicates.

### Chromatin Immunoprecipitation (ChIP) Assay

ChIP assay was performed as described previously (19). Briefly, AGS cells transfected Flag-HOXA13 was fixed with 1% formaldehyde to crosslink DNA and proteins. Chromatin was sonicated to shear DNA to 200–1,000 bp size and incubated with IgG (Sangon, Shanghai, China) or anti-Flag (Cell Signaling Technology). After reversing the protein-DNA cross linking, purified DNA was used to detect the possible binding sites of HOXA13 in promoter region of ABCC4 by agarose gel electrophoresis. The primers were listed below: Primer 1 F: 5’-ACAGAGCCTCACTATGCTGGC-3’, R: 5’-CCTTAACAAGGTCAGCAGCTGC-3’; Primer 2 F: 5’-CCAGCCTGGGCAACAAAGTG-3’, R: 5’-CCACCACACCCGGCTCATAT-3’; Primer 3 F: 5’-AGCCTGGAACTCCTGGGCTAA-3’, R: 5’-TTGATAATTTCCCATGTATATTT-3’; Primer 4 F: 5’-AAAGAAAACCAAATTCTCAAA-3’, R: 5’-AATCCTCCCAACTCAGTTTAAG-3’.

### *In Vivo* Xenograft Model

GC cells (5 × 10^6^) were subcutaneously injected into the back of BALB/c male mice. When the volume of xenografts reached approximately 100 mm^3^, mice were randomly divided into two treatment groups (n = 3): the 5-FU-treated group (shNC + 5-FU and shHOXA13 + 5-FU) and the untreated control group (shNC + CON and shHOXA13 + CON). 5-FU (20 mg/kg) was intraperitoneally injected three times a week for 2 weeks in the treated group and the untreated control group receiving PBS according to the same schedule. Then all mice were euthanized. Tumor volume was calculated by the following formula: V = length × width^2^ × 0.5. All animal studies were approved by Animal Care and Use Committee of Shanghai General Hospital.

### Immunohistochemical Staining (IHC)

IHC assay was conducted as described previously (17). Briefly, the tumor sections were deparaffinized and rehydrated before boiling in sodium citrate solution (0.01 M, pH 6.0) for antigen retrieval. After blocking endogenous peroxidase activity using 3% hydrogen peroxide, the slices were incubated with anti-HOXA13 (1:100; Abcam), anti-ABCC4 (1:100; Abcam), and anti-cleaved caspase-3 (1:100; Affinity, OH, USA) overnight 4°C. After incubation with the suitable secondary antibody, slides were counterstained with hematoxylin.

### Luciferase Reporter Assay

The binding and mutant sequences of HOXA13 3’-UTR were respectively inserted into pGL3 luciferase vector (Genomeditech). Then, the plasmids were co-transfected with miR-139-5p mimics or mimics NC into HEK-293T cells. After a 48-h incubation, the relative luciferase activities were examined using Dual luciferase Assay System (Promega, WI, USA).

### Statistical Analysis

Statistical analyses were conducted using SPSS 22.0 or GraphPad Prism software. The data were presented as the mean ± SD. Comparisons between two groups were performed by Student’s t-test. The correlation of the mRNA expression levels was analyzed using Pearson’s test. P <0.05 was considered statistically significant.

## Results

### High Expression of HOXA13 Is Associated With Poor 5-FU Treatment Response in GC

**Figure 1:**
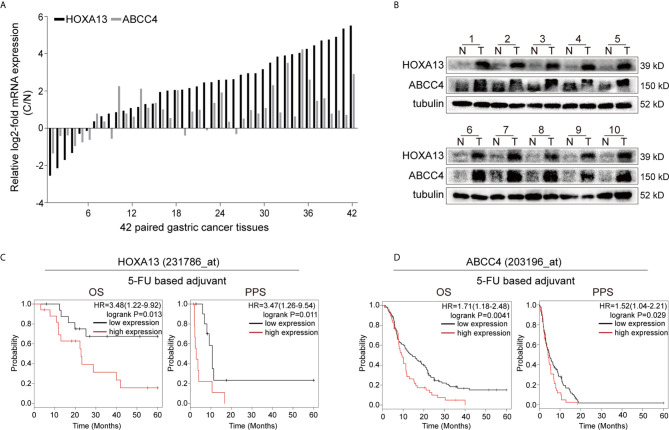
High HOXA13 expression is associated with 5-FU resistance. **(A)** qRT-PCR analysis of HOXA13 and ABCC4 expression in GC tissues compared with paired normal tissues. **(B)** Western blot analysis of HOXA13 and ABCC4 expression in GC tissues compared with paired normal tissues. **(C)** The Kaplan–Meier plotter showed that upregulation of HOXA13 was significantly associated with lower OS and PPS in GC patients with 5-FU treatment. **(D)** In 5-FU based chemotherapy, GC patients with high ABCC4 expression had poorer prognosis (http://kmplot.com/analysis/).

Our previous study revealed that HOXA13 was elevated in GC samples. To confirm the results, qRT-PCR was conducted and showed that the expression of HOXA13 was upregulated in 85.71% (36/42) GC tissues (**Figure 1A**). Correspondently, the protein levels of HOXA13 were increased in GC tissues compared with matched normal tissues (**Figure 1B**). To clarify the clinical significance of HOXA13 in human GC, we analyzed the data in the Kaplan–Meier plotter. As shown in **Figure 1C**, high HOXA13 expression was correlated with poorer OS and PPS in the patients with 5-FU based chemotherapy. These findings suggested that HOXA13 might be associated with poor 5-FU chemotherapy response. However, the worse efficacy of chemotherapy usually involves multiple factors, among which chemoresistance is one of the most common causes. Thus, we hypothesized that HOXA13 played a role in GC resistance to 5-FU and identified it for further investigation.

### HOXA13 Enhances 5-FU Resistance in GC Cells

**Figure 2:**
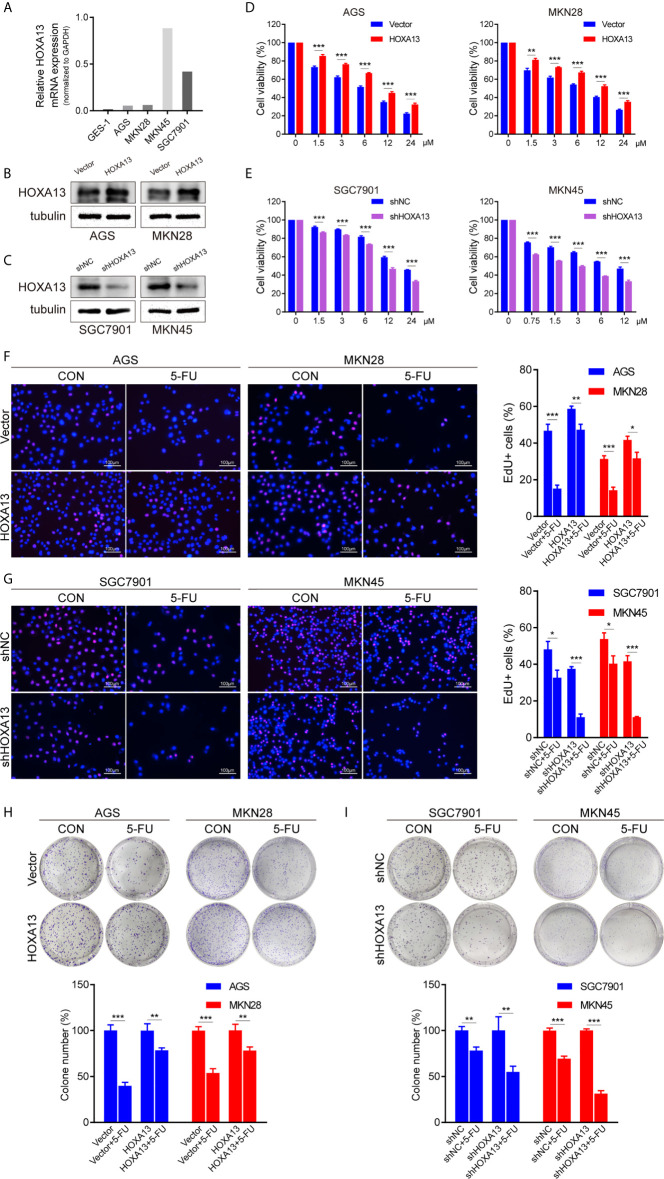
HOXA13 promotes 5-FU resistance in GC cells. **(A)** Relative expression levels of HOXA13 in cell lines were detected by qRT-PCR. **(B, C)** The expression levels of HOXA13 were verified by Western blot in GC cells after transfection. **(D, E)** CCK-8 assays detected relative cell viability of GC cells with various concentrations of 5-FU. **(F G)** The rates of EdU staining in HOXA13+5-FU groups were higher than those of Vector + 5-FU groups, while knockdown of HOXA13 had the opposite effect. Magnification ×200. **(H, I)** After 5-FU treatment, the relative colony formation rates of HOXA13-overexpressing cells were higher than that of Vector groups, while the relative rates of colonies were reduced in HOXA13 knockdown cells. *P < 0.05, **P < 0.01, ***P < 0.001.

To explore the relationship between HOXA13 expression and 5-FU cytotoxic effect on GC cells, we selected AGS and MKN28 to generate stable overexpression cell lines and SGC7901 and MKN45 to generate stable knockdown cell lines, respectively (**Figures 2A–C**, **Supplementary Figures 1A**, **B**). The cytotoxicity of gradient concentrations of 5-FU was detected by CCK-8 assays. As shown in **Figures 2D** and **E**, overexpression of HOXA13 enhanced AGS and MKN28 cells resistance to 5-FU. Conversely, knockdown of HOXA13 decreased 5-FU resistance in SGC7901 and MKN45 cells. In addition, we examined the effects of HOXA13 on cell proliferation in condition of 5-FU. EdU assays indicated that HOXA13-overexpressing cells displayed less significant 5-FU inhibition than the Vector cells did, while HOXA13 knockdown cells showed the opposite (**Figures 2F, G**). Consistently, HOXA13 overexpression cells had relatively higher colony survival rates compared to Vector groups, when treated with 5-FU for colony formation. On the contrary, the colony number of HOXA13-silencing groups was less than that of shNC groups (**Figures 2H, I**). These results indicated that HOXA13 overexpression enhanced 5-FU resistance, reducing the cellular 5-FU sensitivity.

### HOXA13 Knockdown Exacerbates 5-FU-Induced Apoptosis in GC Cells

**Figure 3:**
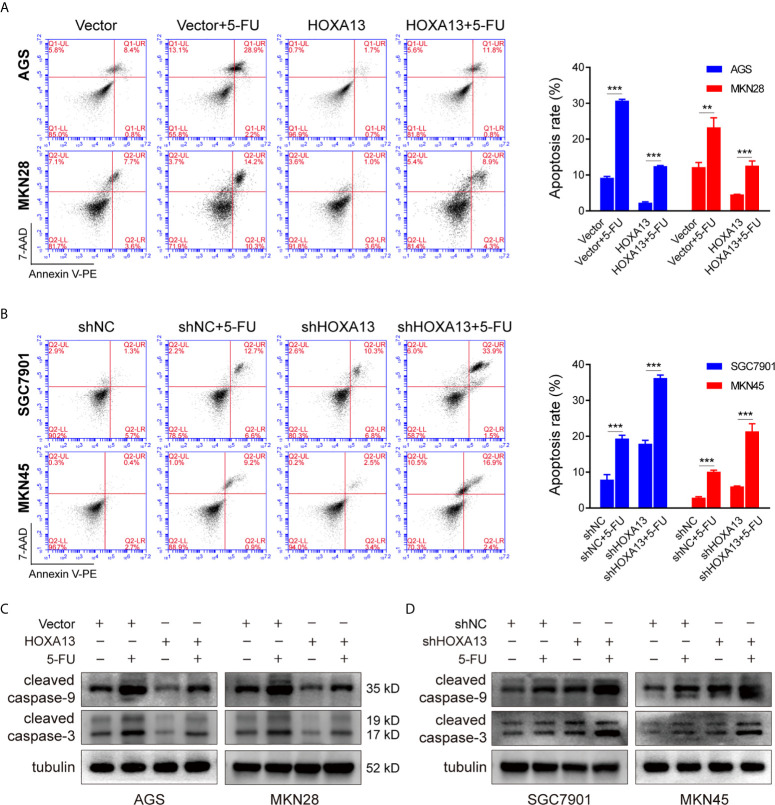
HOXA13 knockdown exacerbates apoptosis induced by 5-FU in GC cells. **(A, B)** Flow cytometry assays detected the effect of altered HOXA13 expression and 5-FU treatment on GC cells apoptosis. **(C, D)** The protein levels of cleaved caspase-9 and cleaved caspase-3 in GC cells were determined by Western blot. **P < 0.01, ***P < 0.001.

Inducing tumor cell apoptosis is considered a critical mechanism of chemotherapy (20). We used flow cytometry to study the effect of HOXA13 on 5-FU-induced apoptosis ability. Compared with Vector group, overexpression of HOXA13 weakened the capacity of 5-FU inducing apoptosis (**Figure 3A**). On the other hand, the apoptosis rates were significantly increased after knockdown of HOXA13 with 5-FU treatment (**Figure 3B**). Additionally, we analyzed the levels of apoptosis-related proteins by Western blot. As predicted, the results of 5-FU treatment showed lower levels of cleaved caspase-9 and cleaved caspase-3 in HOXA13 overexpressing-cells, as well as higher expression levels in HOXA13 knockdown cells (**Figures 3C, D**). The above results revealed that downregulation of HOXA13 expression exacerbated the apoptosis-inducing effect of 5-FU.

### HOXA13 Upregulates ABCC4 Expression *Via* Binding to its Promoter Region

**Figure 4:**
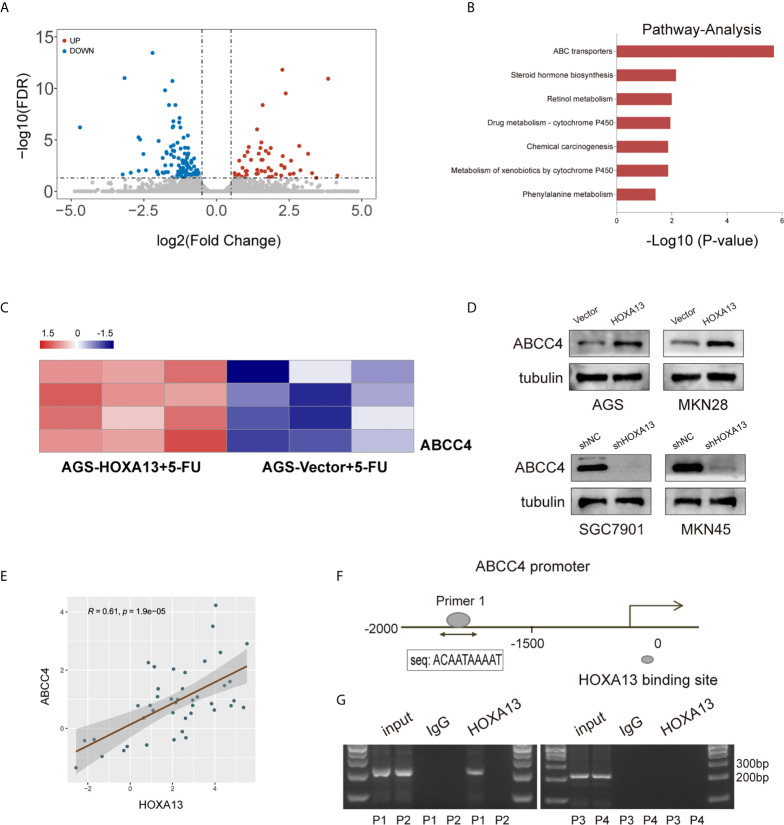
HOXA13 upregulates ABCC4 expression *via* binding to its promoter region. **(A)** Volcano plot showed significantly upregulated genes in AGS-HOXA13 + 5-FU relative to AGS-Vector + 5-FU. **(B)** Pathway analysis revealed that ABC transporter pathway was significantly enriched. **(C)** Heatmap showed that ABCC4 was obviously upregulated in ABC transporter family. **(D)** The protein expression levels of ABCC4 in the indicated cell lines with altered HOXA13 expression. **(E)** Pearson’s correlation analysis of the mRNA levels of HOXA13 and ABCC4 in GC samples. **(F)** HOXA13 binding to the ABCC4 promoter region was predicted by JASPAR (http://jaspar.genereg.net/). **(G)** The results of ChIP assay and agarose gel electrophoresis indicated HOXA13 enriched in primer 1 within the ABCC4 promoter.

To elucidate the underlying mechanism of HOXA13-mediated 5-FU resistance in GC cells, we performed RNA sequencing to compare the transcriptional alterations of AGS-HOXA13 + 5-FU and AGS-Vector + 5-FU cells. The volcano plot indicated 64 upregulated genes and 121 downregulated genes in the AGS-HOXA13 + 5-FU group (Fold change >1.5, P <0.05, **Figure 4A**). Subsequently, we performed pathway analysis based on the KEGG database and found that the upregulated genes were significantly relevant to ABC transporters (**Figure 4B**). Due to the potential clinical significance of ABC transporters in chemoresistance (21, 22), we postulated that ABC transporters activation might play an important role in HOXA13-mediated 5-FU resistance. Further analyzing the relationship between HOXA13 and ABC transporters, we found upregulation in transcript amounts of four ABC transporter genes, ABCC4, ABCA5, ABCA8 and ABCA12, detected in the AGS-HOXA13 cells treated by 5-FU, among which the differential expression of ABCC4 was prominent (**Figure 4C**). Subsequently, we examined ABCC4 expression in GC cells with different HOXA13 expression. It showed that the significant increase in ABCC4 expression was accompanied by elevated level of HOXA13. Likewise, in SGC7901 and MKN45 cells, ABCC4 downregulation was linked to HOXA13 knockdown (**Figure 4D**). ABCC4 was upregulated in 76.19% (32/42) GC samples indicated by qRT-PCR (**Figure 1A**), and positively correlated with HOXA13 in mRNA levels disclosed by the correlation analysis (**Figure 4E**). The patients with high ABCC4 expression had shorter OS and PPS with treatment of 5-FU shown by the Kaplan–Meier plotter (**Figure 1D**). To explore the relationship between HOXA13 and ABCC4, we predicted the binding sites of HOXA13 in ABCC4 promoter region by JASPAR (http://jaspar.genereg.net/) and designed four primer sequences (**Supplementary Figure 1C**). HOXA13 was demonstrated to enriched in primer 1 within the ABCC4 promoter tested by ChIP assay and agarose gel electrophoresis (**Figures 4F, G**). These results indicated that HOXA13 might upregulate ABCC4 expression *via* binding to its promoter region.

### siABCC4 Reverses HOXA13-Induced 5-FU Resistance in GC Cells

**Figure 5:**
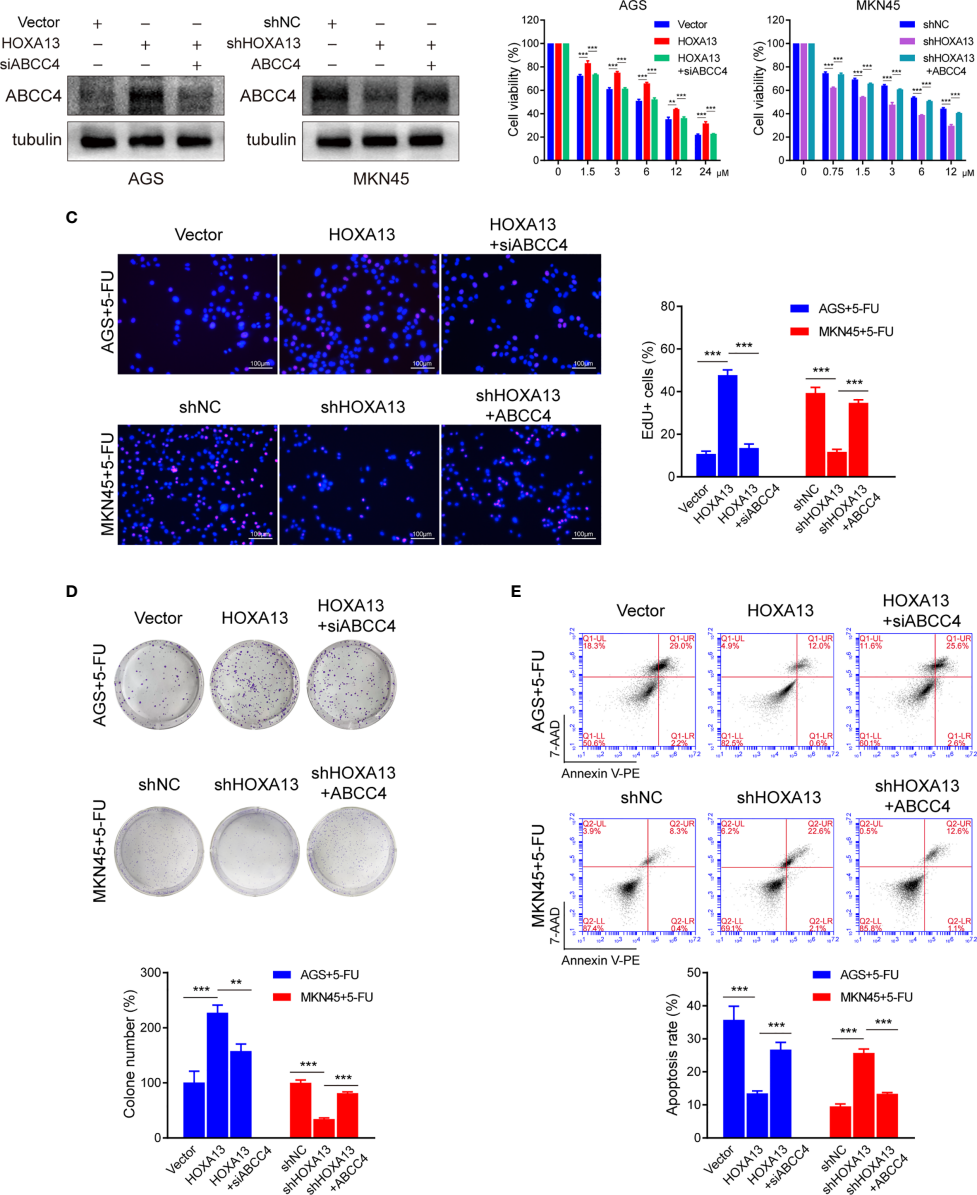
siABCC4 reverses HOXA13-mediated 5-FU resistance in GC cells. **(A)** The protein levels of ABCC4 were detected in AGS cells including Vector, HOXA13 and HOXA13 + siABCC4 groups and MKN45 cells including shNC, shHOXA13 and shHOXA13 + ABCC4 groups. **(B–D)** CCK-8 assays, EdU assays and colony formation assays revealed that depletion of ABCC4 enhanced anti-proliferative effect of 5-FU in HOXA13-overexpressing cells, while overexpression of ABCC4 weakened that of 5-FU in HOXA13 knockdown cells. Magnification ×200. **(E)** After inhibiting of ABCC4 expression, the apoptotic levels of HOXA13-overexpressing cells induced by 5-FU was increased, while ABCC4 overexpression in HOXA13 knockdown cells had the same rescue effect. **P < 0.01, ***P < 0.001.

To further investigate the role of ABCC4 in HOXA13-mediated chemoresistance, we used siRNA to silence ABCC4 expression in AGS-HOXA13 cells. Also, MKN45-shHOXA13 cells were transiently transfected with ABCC4-overexpressing plasmid (**Figure 5A**). Upregulating ABCC4 expression reversed partly the effects of HOXA13 knockdown on 5-FU anti-proliferation process, while decreasing ABCC4 expression, the cell proliferation inhibitory effects of 5-FU were restored, indicated by CCK-8, EdU and colony formation assays (**Figures 5B–D**). In addition, after downregulating ABCC4, the apoptotic rate of AGS-HOXA13 cells partly increased suggested by flow cytometry. Conversely, in MKN45-shHOXA13 cells, upregulation of ABCC4 produced the same rescue effect (**Figure 5E**). Overall, the results demonstrated that HOXA13 promoted 5-FU resistance of GC cells through upregulating ABCC4 expression.

### HOXA13 Knockdown Sensitizes GC Cells to 5-FU *In Vivo*

**Figure 6:**
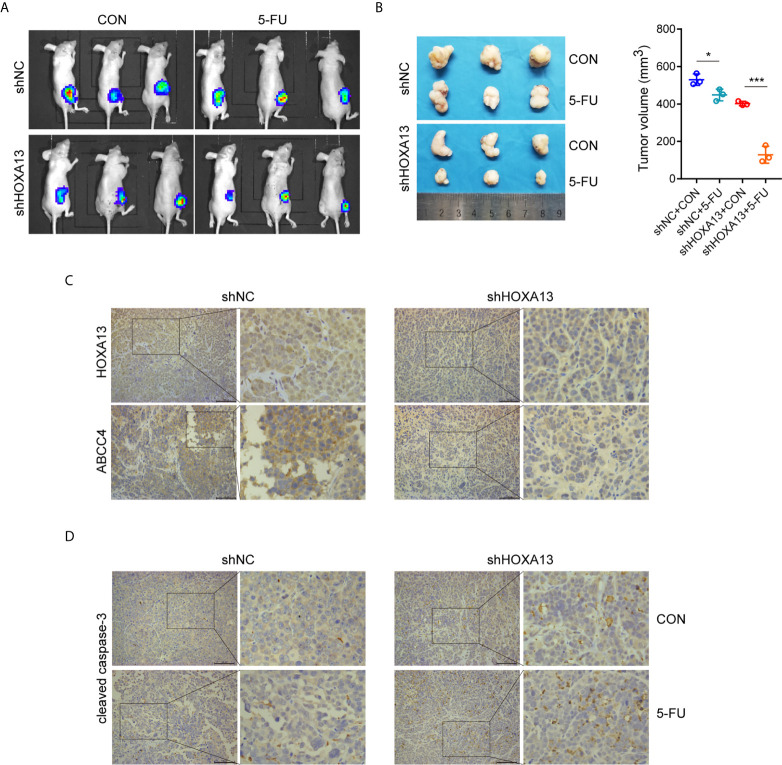
HOXA13 knockdown increases sensitivity of GC cells to 5-FU *in vivo*. **(A)** Bioluminescence images of tumors formed by subcutaneously injecting MKN45 cells, followed by 5-FU or control (CON) treatment. **(B)** The final tumor volumes in each group were measured. **(C)** IHC staining of HOXA13 and ABCC4 were performed in tumor tissues. **(D)** IHC staining of cleaved caspase-3 was obvious in shHOXA13 + 5-FU group. Magnification ×200. *P < 0.05, ***P < 0.001.

We generated a subcutaneous tumor model to assess the role of HOXA13 in 5-FU anti-tumor effect *in vivo*. The result showed that the tumor volumes of MKN45-shHOXA13 group were smaller than those of shNC group, indicating knockdown of HOXA13 weakened tumorigenicity of MKN45 cells. Even more remarkably, although the tumor sizes of 5-FU groups were smaller than those of CON groups, 5-FU impeded tumor formation of shHOXA13 group more significantly (402.19 to 128.92 mm^3^; −67.95%), compared with shNC group (529.75 to 448.38 mm^3^; −15.36%), suggesting that suppression of HOXA13 improved the sensitivity of MKN45 cells to 5-FU (**Figures 6A, B**). The positive staining of HOXA13 and ABCC4, shown by IHC, was detected in shNC group, whereas the expression of ABCC4 was reduced in shHOXA13 group (**Figure 6C**). When comparing four groups of xenograft tumors (shNC + CON, shNC + 5-FU, shHOXA13 + CON and shHOXA13 + 5-FU), it showed that the staining intensity of cleaved caspase-3 was the lowest in shNC + CON group, mild in shNC + 5-FU and shHOXA13 + CON groups, and the most significant in shHOXA13 + 5-FU group (**Figure 6D**).

### HOXA13 Is Directly Targeted by miR-139-5p

**Figure 7:**
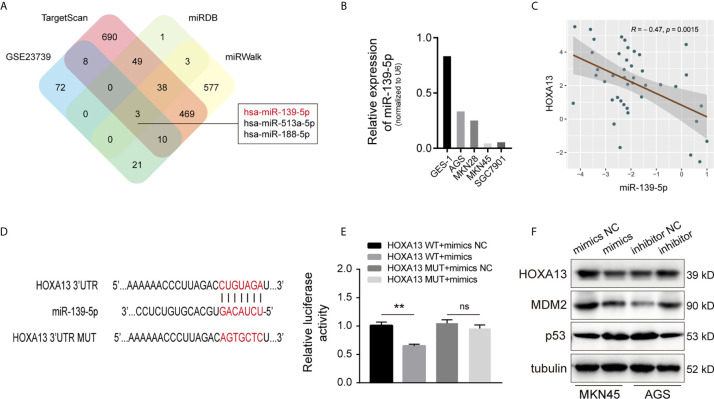
HOXA13 is directly targeted by miR-139-5p in GC. **(A)** Potential miRNAs that target HOXA13 were predicted by GEO dataset and online prediction tools. **(B)** Relative expression levels of miR-139-5p in cell lines were detected by qRT-PCR. **(C)** Pearson’s correlation analysis of the mRNA levels of miR-139-5p and HOXA13 in GC samples. **(D)** The predicted miR-139-5p binding site in HOXA13 and sequences of wild-type (WT) and mutant (MUT) 3’-UTR of HOXA13. **(E)** Relative luciferase activity were performed in HEK-293T cells after co-transfection with HOXA13 WT or HOXA13 MUT and miR-139-5p mimics or NC. **(F)** The protein expression levels of HOXA13, MDM2 and p53 were detected in MKN45 cells transfected with miR-139-5p mimics or NC and AGS cells transfected with miR-139-5p inhibitor or NC. **P < 0.01. ns, no significant.

MicroRNAs (miRNAs), a class of small non-coding RNAs, can regulate the expression of target mRNAs by interacting with the 3’-UTR region (23). To further investigate whether HOXA13 expression can be directly regulated by miRNAs in GC, we analyzed downregulated miRNAs in microarray expression dataset (GSE23739). Combined with bioinformatics analysis (TargetScan, miRDB and miRWalk), we discovered that three candidate miRNAs potentially bound with HOXA13 (**Figure 7A**). Among these miRNAs, only miR-139-5p was involved in the progression of gastric cancer according to previous reports (24–26), which was further verified downregulated in GC cells compared to GES-1 and negatively associated with the expression of HOXA13 in GC tissues in our study (**Figures 7B, C**). Therefore, miR-139-5p was selected as a putative candidate for further validation. The luciferase reporter assay of HEK-293T cells showed that fluorescence activity was significantly reduced after co-transfection of miR-139-5p mimics and wild-type HOXA13, while co-transfection of miR-139-5p mimics and mutant HOXA13 had no effect on luciferase activity (**Figures 7D, E**). Meanwhile, miR-139-5p mimics transfection decreased the protein level of HOXA13 compare to that in mimics NC, whereas transfecting miR-139-5p inhibitor led to a converse response (**Figure 7F**). Previous research revealed that HOXA13 conferred 5-FU resistance *via* MDM2-p53 pathway (27). In this study, we observed that MDM2 expression was decreased with downregulation of HOXA13 transfected with mimics, while p53 expression was increased (**Figure 7F**). Taken together, these findings indicated that miR-139-5p could directly target HOXA13 in GC.

## Discussion

HOXA13 has been reported to play a pivotal role in the normal growth and differentiation of mammalian tissues (28). Recently, a booming number of studies have demonstrated that aberrant HOXA13 expression correlates with proliferation, metastasis, prognosis and chemoresistance in various types of cancer (29–31). High expression of HOXA13 was an independent prognostic marker of poor outcome in GC elucidated in our previous study (32). HOXA13 overexpression promoted the growth and metastasis of GC cells (17). Herein, we further explore the role and mechanism of HOXA13 in chemosensitivity of GC.

In the present study, we reconfirmed that HOXA13 was upregulated in GC samples. Next, we analyzed the prognosis of GC patients receiving 5-FU based chemotherapy. And the Kaplan–Meier plotter suggested that high expression of HOXA13 was associated with poor response of 5-FU treatment in GC. However, whether the unfavorable prognosis of 5-FU treatment in GC was directly attributed to chemoresistance required detailed validation.

In order to confirm the possibility of the hypothesis, we examined that whether altered HOXA13 expression had influence on 5-FU sensitivity of GC cells. The results showed that HOXA13 overexpression promoted GC cells to be resistant to 5-FU, whereas 5-FU resistance of HOXA13 knockdown groups significantly diminished compared with that of shNC groups, indicating that HOXA13 upregulation enhanced 5-FU resistance, namely weakened sensitivity of GC cells to 5-FU.

Subsequently, we observed the effect of HOXA13 expression on GC cell growth with 5-FU treatment. Cells in each group with low expression of HOX13 treated with 5-FU showed the slowest proliferation rate and smallest colony ratio, demonstrated by EdU staining and colony formation assay respectively. Afterward, we used flow apoptosis assay to examine the proportion of apoptotic cells in GC cells upon 5-FU treatment. The results showed that the apoptotic rate of shHOXA13 + 5-FU groups was significantly increased compared with shNC+5-FU groups, suggesting that HOXA13 knockdown enhanced 5-FU-induced apoptosis. The above experiments indicated that HOXA13 knockdown enhanced the inhibition effect of 5-FU on cell proliferation and promoted 5-FU-induced apoptosis, thereby increasing the sensitivity of GC cells to 5-FU.

*In vivo* experiment also verified the above results. Compared with shNC group, the tumor sizes of shHOXA13 group were more significantly inhibited by 5-FU, indicating that downregulation of HOXA13 expression improved the sensitivity of GC cells to 5-FU *in vivo*. What’s more, the expression of cleaved caspase-3 in shHOXA13 + 5-FU group was significantly higher than other three groups, suggesting that HOXA13 knockdown augmented 5-FU induced apoptosis *in vivo*.

To explore the underlying mechanisms of HOXA13-mediated 5-FU resistance in GC cells, transcriptome sequencing was utilized to profile differentially expressed genes in AGS cells with 5-FU treatment (AGS-HOXA13 + 5-FU *vs*. AGS-Vector + 5-FU). The results showed that in AGS-HOXA13 + 5-FU group, upregulated genes were predominantly enriched in the following pathways: ABC transporters, drug metabolism-cytochrome P450 and chemical carcinogenesis, among which the enrichment of ABC transporters dominated. To date, ample studies have demonstrated that a major mechanism of chemoresistance in cancers is the upregulation of ABC transporters expression (33, 34). ABC transporters, located in cell membrane, are a group of ATP-dependent pumps that transports substrates out of cells (35). Of these, the C subgroup, also called the multidrug resistance-associated proteins (MRPs), has attracted growing attention in tumor chemoresistance (36, 37). Combined with the sequencing results, we speculated that 5-FU resistance induced by HOXA13 might be related to activation of ABC transporters. Further analysis confirmed that ABCC4 was significantly upregulated in AGS-HOXA13+5-FU cells leading to the inference that ABCC4 might be a potential downstream target of HOXA13. As a member of MRPs, ABCC4 is a versatile efflux transporter for many drugs, including chemotherapeutic drugs (38). As shown by research in prostate cancer, inhibition of ABCC4 expression restores the docetaxel sensitivity (39). ABCC4 is transcriptional regulated by FoxM1, promoting carboplatin resistance in retinoblastoma (40). Abbaszadegan et al. found that KCTD12 decreases 5-FU resistance in esophageal squamous carcinoma cell by down-regulating ABCC4 (41). Interestingly, this study revealed that ABCC4 was upregulated in GC tissues, and mRNA expression of HOXA13 was positively correlated with that of ABCC4. The unfavorable prognosis of GC patients with high ABCC4 expression was found in the case of 5-FU based chemotherapy, suggesting that ABCC4 expression was associated with efficacy of 5-FU in GC patients.

To further investigate whether there was a regulatory relationship between HOXA13 and ABCC4, we examined the impact of HOXA13 expression alternation on ABCC4 in GC cells. The results showed that ABCC4 expression was upregulated in HOXA13-overexpressing cells and downregulated in HOXA13 knockdown cells, prompting that HOXA13 might modulate the expression of ABCC4. Noticeably, the JASPAR database indicated the possibility of HOXA13 binding to the ABCC4 promoter. Therefore, we designed four primer sequences for ChIP assay and studied whether HOXA13 could bind to promoter region of ABCC4. The result showed that HOXA13 might enrich in the ABCC4 promoter region. Subsequent rescue experiments confirmed that inhibition of ABCC4 expression attenuated the ability of HOXA13 overexpression enhanced 5-FU resistance of GC cells, while upregulation of ABCC4 partly reversed the process of HOXA13 knockdown promoted GC cells sensitivity to 5-FU. These findings suggested that HOXA13 upregulated ABCC4 expression possibly by binding to its promoter, and ABCC4 might play a crucial role in HOXA13-mediated insensitivity of GC to 5-FU.

Increasing evidences have demonstrated that miRNAs play an important role in tumor progression through post-transcriptionally regulating functional mRNAs expression (42). In this study, miR-139-5p, identified by GEO dataset and bioinformatics analyses, was downregulated in GC cells and negatively correlated with HOXA13 in GC tissues. Moreover, by mechanism experiments, we confirmed that miR-139-5p directly might bind to HOXA13 3’-UTR to downregulate its expression. However, the role of miR-139-5p in chemoresistance of GC cells remains to further researched.

In conclusion, our study shows that HOXA13 is upregulated in GC samples and associated with poor prognosis of GC patients in the case of 5-FU treatment. High HOXA13 expression enhances 5-FU resistance and reduces 5-FU sensitivity, as well as alleviates the anti-proliferative effect of 5-FU and suppresses 5-FU-induced cell apoptosis. And ABC transporter pathway activation, especially ABCC4 upregulation, may play an important role in HOXA13-mediated 5-FU resistance. HOXA13 expression is directly suppressed by miR-139-5p in GC cells. Targeting the HOXA13/ABCC4 axis is expected to be a potential therapeutic strategy for reducing resistance to chemotherapy.

## Data Availability Statement

The RNA sequencing data are available on Figshare (https://figshare.com/articles/dataset/RNA-seq_AGS-HOXA13_5-FU_vs_AGS-Vector_5-FU_All_xlsx/14546811).

## Ethics Statement

The studies involving human participants were reviewed and approved by Shanghai General Hospital. The patients/participants provided their written informed consent to participate in this study. The animal study was reviewed and approved by Shanghai General Hospital.

## Author Contributions

ZC, ZQ, and XC designed and performed the experiments. LL and QW performed animal experiments. ZC and ZQ analyzed the data and wrote the manuscript. XC supervised the project. All authors contributed to the article and approved the submitted version.

## Conflict of Interest

The authors declare that the research was conducted in the absence of any commercial or financial relationships that could be construed as a potential conflict of interest.

## Supplementary Material

Supplementary Figure 1
**(A)** Establishment of HOXA13-overexpressing cell lines in AGS and MKN28 cells, confirmed by qRT-PCR. **(B)** Establishment of HOXA13 knockdown cell lines in SGC7901 and MKN45 cells, confirmed by qRT-PCR. **(C)** The binding sites of HOXA13 in ABCC4 promoter region were predicted by JASPAR database and primer sequences were designed. ****P* < 0.001.
