# Supplementary figures and images for: HOXA13, Negatively Regulated by miR-139-5p, Decreases the Sensitivity of Gastric Cancer to 5-Fluorouracil Possibly by Targeting ABCC4
Source: Front Oncol. 2021 May 21;11:645979. doi: 10.3389/fonc.2021.645979 (PMC8175971; doi:10.3389/fonc.2021.645979)

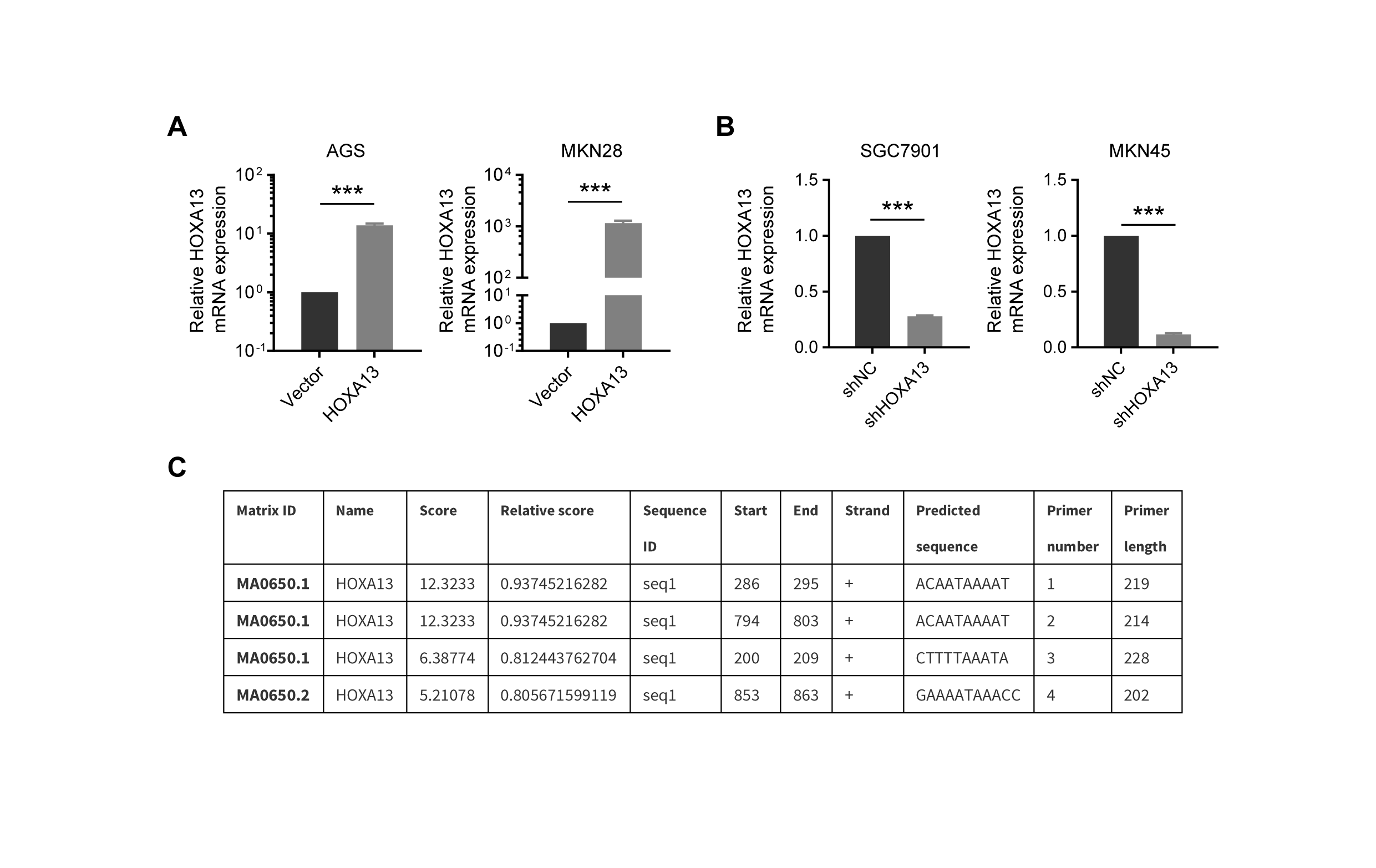

Supplement: Supplementary Figure 1 — (A) Establishment of HOXA13-overexpressing cell lines in AGS and MKN28 cells, confirmed by qRT-PCR. (B) Establishment of HOXA13 knockdown cell lines in SGC7901 and MKN45 cells, confirmed by qRT-PCR. (C) The binding sites of HOXA13 in ABCC4 promoter region were predicted by JASPAR database and primer sequences were designed. ***P < 0.001. [file Image_1.tif]
